# Negative pressure wound therapy induces early wound healing by increased and accelerated expression of vascular endothelial growth factor receptors

**DOI:** 10.1007/s00238-016-1200-z

**Published:** 2016-06-13

**Authors:** Tsuruhito Tanaka, Nirmal Panthee, Yoshifumi Itoda, Naoko Yamauchi, Masashi Fukayama, Minoru Ono

**Affiliations:** 1 Department of Cardiac Surgery, The University of Tokyo, 7-3-1, Hongo Bunkyo-ku, Tokyo, Japan; 2 Department of Pathology, The University of Tokyo, 7-3-1, Hongo Bunkyo-ku, Tokyo, Japan

**Keywords:** Vascular endothelial growth factor receptor (VEGFR), Negative pressure wound therapy (NPWT), Wound-healing, Immunohistochemistry

## Abstract

**Background:**

Negative pressure wound therapy (NPWT) is commonly used to accelerate wound healing, especially following thoracic surgery; however, the mechanism remains elusive. Given the important role of vasculogenesis in wound healing, we evaluated whether NPWT might accelerate vasculogenesis in the wound area. Toward this end, we investigated the temporal expression of vascular endothelial growth factor receptors (VEGFRs) in an NPWT-wound healing rabbit model.

**Methods:**

Rabbits were divided into an NPWT group and a non-NPWT control group, and tissue samples were collected around wounds made in the skin of each rabbit at five time points: 0, 7, 14, 21, and 28 days after wound creation. Cryopreserved samples were then immunostained and subject to image analysis to evaluate the temporal changes in VEGFR1, VEGFR2, and VEGFR3 expression in the wound-healing process.

**Results:**

Results of histological analysis of the temporal changes in VEGFR expression throughout the healing process showed that compared to the control group, VEGFR2 and VEGFR3 were abundantly and rapidly expressed in the NPWT group, and were expressed earlier than VEGFR1.

**Conclusions:**

NPWT promotes the expression of VEGFR2 and VEGFR3, which provides insight into the mechanism by which NPWT accelerates wound healing.

Level of Evidence: Not ratable.

## Introduction

Negative pressure wound therapy (NPWT) is a general technique used to accelerate wound healing with controlled application of sub-atmospheric pressure to the wound. NPWT is commonly used for postoperative mediastinitis and similar conditions in the field of thoracic surgery; however, the mechanism by which it exerts its positive effects remains poorly understood [1, 2].

Extensive research has been conducted on approaches to achieve rapid wound healing of surgical incisions, including methods for promoting the early formation of granulation tissue and associated ectoderm-derived skin and mesoderm-derived vascular connective tissues such as the dermis and subcutaneous tissue, via accelerating initial vasculogenesis in the wound area [3–7].

Vasculogenesis is a common process required for the regeneration of all tissue types.

The peptides and proteins that are acquired by the endothelial cells during the differentiation and generation of these vascular systems have been characterized, and these same factors are believed to be expressed both in the cell and on the cell membrane surface for the regeneration and creation of the vascular system in wound healing. One of the main factors contributing to these processes is vascular endothelial growth factor (VEGF) [8–15].

VEGF exists in various forms, including VEGF-A, VEGF-C, and VEGF-D, which are all involved in the regeneration and differentiation of the vascular system. VEGF-C and VEGF-D act together to form a signaling cascade to promote VEGF receptor 2 (VEGFR2), and VEGF-C acts alone on neuropilin-2 (Nrp2), which also functions as a nerve growth factor. VEGF-A also encompasses the subsets VEGF-A12 and VEGF-A165, which bind to VEGFR1 and VEGFR2. VEGFR1 and VEGFR2 are generally considered as receptors involved in vasculogenesis and angiogenesis, whereas VEGFR2 and VEGFR3 are regarded as receptors involved in lymphangiogenesis [16–20].

One of the proposed mechanisms of NPWT is the induction of the blood vessel growth factors required for wound healing; in particular, Kinetic Concept Inc.’s Vacuum-Assisted Closure Advanced Therapy System (KCI-VAC ATS) has been widely used for wound healing in clinical settings. The benefits of this system are thought to result from local stimulation of porous urethane that is in contact with the wound surface, and the creation of a negative-pressure environment enclosing the entire wound; indeed, various reports have stated that these two processes act to induce the revascularization factors involved in functional recapillarization of the wound surface [21–25]. There are also reports that NPWT leads to vascular induction from macrophage-induced fibroblast cells via the promotion of immune system factors at the wound surface [26, 27]. However, these NPWT actions have been observed in only a piecemeal manner, and the specific effects of NPWT on the formation of new blood vessels and new lymphatic vessels during wound healing have not yet been clarified. Therefore, in this study, we investigated the specific mechanism by which NPWT might promote the wound-healing process with a focus on the actions of VEGFRs involved in signaling cascades with VEGF-C and VEGF-D, important growth and differentiation factors of the vascular system.

## Materials and methods

### Animals

Healthy male New Zealand White rabbits (Tokyo Laboratory Animals Science, Tokyo, Japan) weighing 3.00–3.49 kg (mean 3.25 kg) were divided into an NPWT group (*n* = 7) that underwent NPWT (with the KCI VAC-ATS system, catalog no. M6257755.R, San Antonio, TX, USA) and a control group (*n* = 7) that only received gauze protection. All animals were maintained in an experimental rearing house (E207) at the University of Tokyo Graduate School of Agricultural and Life Sciences laboratory building. The rabbits were allowed to acclimatize for 1 week before experimentation and were fed 150 g/day of LRC4 (Oriental Yeast, Co., Tokyo, Japan).

### Forming the wound surface and NPWT

**Fig. 1:**
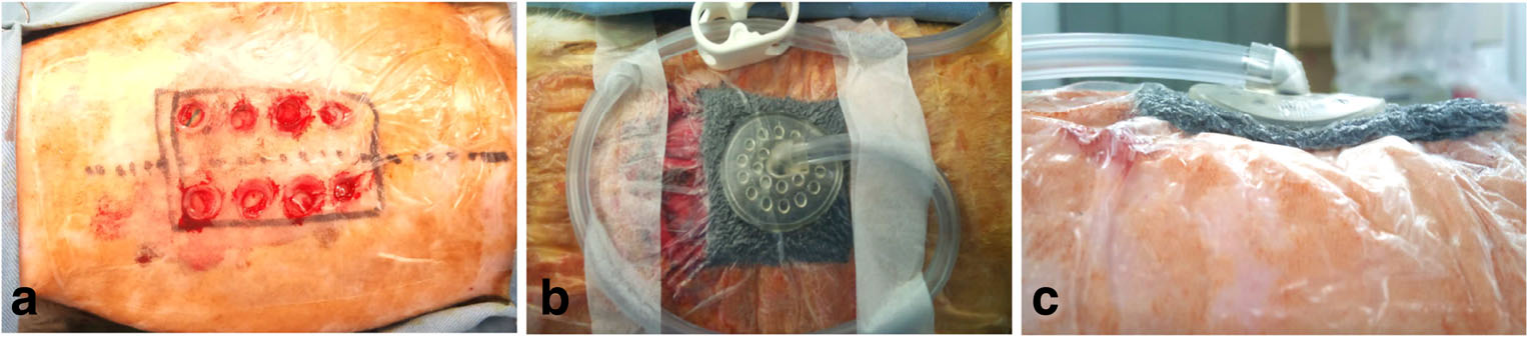
**a** Injured wound surfaces of the back skin of a rabbit under anesthesia at day 0 (control and NPWT group). **b** Experimental surface of the rabbit back skin covered and vacuumed by the VAC ATS-system fixed with tape in the NPWT experimental group. **c** Horizontal view of the rabbit back skin surfaces in the NPWT experimental group by the VAC AST-system with a negative pressure of 125 mmHg

A mixture of 1.5 mL of ketamine and 1.0 mL of xylazine was injected intramuscularly and guided under very deep anesthesia prepared as a 2.5-mL solution comprising 1.5 mL of 500 mg/10 mL of Ketalar muscle injections (First Sankyo Propharma, catalog no. 1119400A3026, Tokyo, Japan) and 1.0 mL of Selactar 2 % injections (Bayer Yakuhin, catalog no. DY006197, Tokyo, Japan). Using Biopsy Trepan (Kai Corp., catalog no. BP-80F, Tokyo, Japan), we formed subcutaneous wounds with a length of 7 mm and diameter of 8 mm in eight sites per animal (Fig. 1a). Tissue of the wound surface was collected for day-0 (baseline) samples containing the foam dressing only for both the control group and the NPWT group.

For the control group (normal pressure), all eight wound surfaces were covered with gauze, and on days 7, 14, 21, and 28, a 10-mm square was cut with a scalpel to the left and right of each wound. Seven rabbits were used to evaluate changes in tissue samples over time.

In the NPWT group, the urethane foam that came with the VAC was embedded in the same hole as the wound at the wound site, placed so that half the thickness of the urethane foam covered the entire wound, and then a pressure-sensitive adhesive sheet (drape) was applied (Fig. 1b). The VAC-ATS system (KCI) was started up in continuous mode with a suction pressure of 125 mmHg, which is standard, and the rabbits were maintained in cages. On days 7, 14, 21, and 28, 10-mm squares were cut to the left and right of each wound with a scalpel under deep anesthesia applied in the same manner described above for wound formation. Tissues from the seven rabbits in the NPWT group were used to evaluate changes over time.

### Preparation of tissue sections and immunostaining

Tissue samples from two sites of the wound floor were collected per animal at days 0, 7, 14, 21, and 28, and were cryopreserved in liquid nitrogen in a vacuum refrigerator at −80 °C using an optimal-cutting temperature compound gel (OCT; Sakura Finetek Japan catalog no. 4583, Tokyo, Japan).

The frozen tissue blocks were prepared into 3-μm serial sections using a cryostat (Leica CM1850; Leica Biosystems, Heidelberger, Nussloch, Germany) and were applied to glass slides and naturally dried at room temperature. After 10 min of immersion in 10 % formalin, the slides were washed with tap water and subject to hematoxylin and eosin (HE) staining.

After 10 min of immersion in acetone following the preparation of sections in the same manner as described above using the cryostat, the slides were dried with a dryer and immersed in 10 % normal goat serum (DAKO Co., catalog no. X0907, Glostrup, Denmark) with a saline solution for 20 min at room temperature. The tissue sections were immunostained with the primary antibodies (goat anti-mouse VEGFR1, VEGFR2, and VEGFR3; R&D Systems catalog nos. AF471, AF644, and AF743, respectively, Minneapolis, MN, USA; 1000-fold dilution) for 16 h at 4 °C within a dedicated refrigerator. The slides were then washed with Tris-buffered saline (TBS; Takara Co., catalog no. T903, Tokyo, Japan) and treated with endogenous peroxidase using TBS with 3 % hydrogen peroxide and then reacted with biotin-labeled secondary antibodies (ABC reagent, Vector’s VECTASTAIN Elite ABC Kit; VECTOR LAB, Universal Elite ABC Kit, catalog no. PK-6200, Burlingame, CA, USA) for 30 min. The slides were washed with TBS again, developed with DAB solution, and subjected to nuclear staining with HE.

### Microscopy and image analysis

After washing under running water, the slides were dehydrated with ethanol and xylene and then sealed with a cover glass and observed. To evaluate tissue morphology, ten consecutive tissue sections were HE-stained, sealed, and observed in the same manner.

The stained tissue sections on glass sides were observed at ×400 magnification on an Olympus CX40 microscope, with a UPlanApo lens (×40 and ×10 objectives), and ten consecutive random fields of view were extracted for analysis so that the observer was blinded to the slide identities. The selected fields were imaged with a digital camera (OLYMPUS CAMEDIA C-3030), and the captured JPEG images were analyzed with Photoshop software (Adobe Systems Inc., San Jose, CA, USA) to convert the shading of the degree of DAB staining on the JPEG images into the number of pixels, which represented the overall immunoreactivity or expression level.

### Statistical analysis

The intensity of DAB immunostaining using various antibodies was represented as pixel numbers from the obtained images, and the mean values were compared between groups using Student’s *t* test; *p* <0.01 was considered statistically significant. The data were analyzed within the control group and the experimental group separately, with comparisons in expression levels made between different time points (day 0, 7, 14, 21, and 28). The proportion of the degree of the overall staining for VEGFRs attributed to VEGFR1, 2, and 3 at each time point was also calculated and is referred to as the “occupancy” (%).

## Results

### VEGFR expression during normal-pressure wound healing (control)

**Fig. 2:**
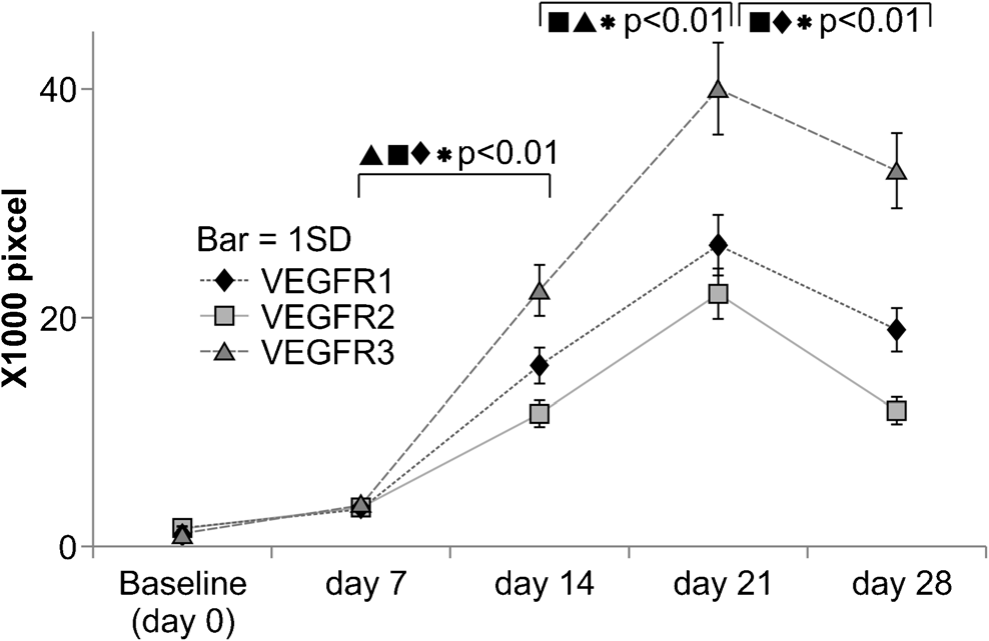
Changes in immunoreactivity in the control group at days 0–28. The *x*-axis indicates the day of measurement after wound creation: day 0 (baseline level), day 7, day 14, day 21, and day 28. The *y*-axis shows values for the immunoreactivity of anti-VEGFR1, anti-VEGFR2, and anti-VEGFR3 antibodies, respectively, converted into pixel numbers (×1000) from the captured digital images. The *bars* indicate 1 SD. Significant differences were determined between days within individual groups (*p < 0.01)

The magnitude (calculated as pixel units) of immunoreactivity to the VEGFRs in the control group is shown in Fig. 2. The expression of VEGFR1 and VEGFR3 increased continuously from day 7 until day 21, but VEGFR2 showed a lower rate of increase from day 7 until day 14. This indicates that different VEGFRs show distinct patterns of expression induction at the initial stages of wound healing (from day 7 to day 14).

Immunoreactivity to anti-VEGFR1, 2, and 3 antibodies did not vary significantly at the initial stages of wound healing from day 0 to day 7. However, significant increases (*p* < 0.01) in immunoreactivity to VEGFR1, 2, and 3 were observed in the later stages of healing, from day 7 to day 21. In particular, the expression of VEGFR3 increased 5.95-fold from day 7 to day 14 and increased 1.8-fold from day 14 to day 21, while the expression of VEGFR1 increased 4.92-fold from day 7 to day 14 and 1.63-fold from day 14 to day 21. There was no significant difference in the rate of increase between VEGFR1 and VEGFR3 in the two time intervals of day 7 to day 14 and day 14 to day 21 (*p* > 0.01). The expression of VEGFR2 increased 3.43-fold from day 7 to day 14 and increased 1.92-fold from day 14 to day 21; VEGFR2 expression was significantly lower than that of VEGFR1 and VEGFR3 from day 7 to day 14 (*p* < 0.01). The expression of all of the VEGFRs decreased from day 21 to day 28; in particular, VEGFR2 and VEGFR3 expression decreased significantly (*p* < 0.01), whereas VEGFR3 did not show a significant decrease (*p* > 0.05). These results support the notion that the three receptors have different roles in the process of differentiation of the vasculature.

### Changes in the occupancy of each VEGFR in the control group during wound healing

**Fig. 3:**
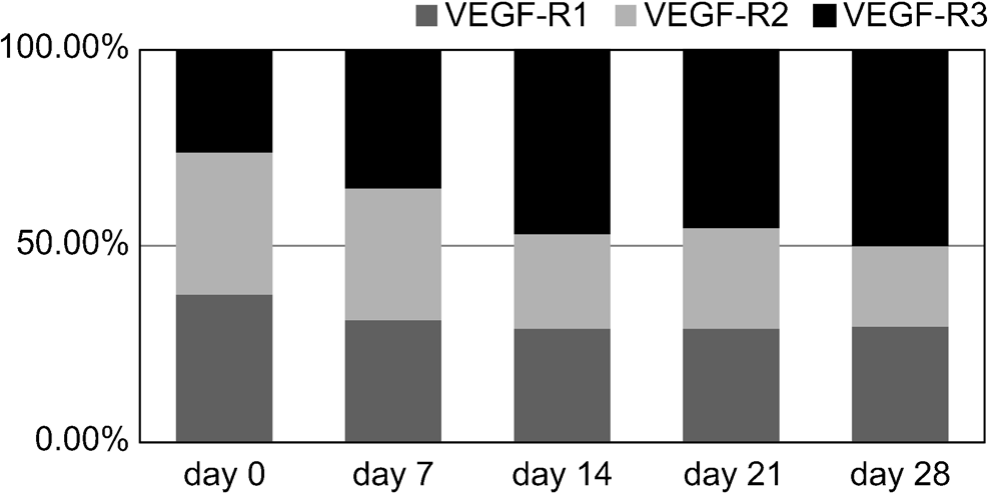
Time course of the occupancy for each VEGFR during normal wound healing. The *x*-axis indicates the day of measurement after wound creation: day 0 (baseline), day 7, day 14, day 21, and day 28. The *y*-axis shows the relative degree of staining of anti-VEGFR1, anti-VEGFR2, and anti-VEGFR3 antibodies in relation to the total stained area of all VEGFRs to reflect the occupancy (%) of VEGFR1, VEGFR2, and VEGFR3, respectively. These values were converted to ×1000 pixels from the captured digital images

The changes in the proportion of VEGFR expression attributed to each of the three receptors (occupancy %) are shown in Fig. 3. VEGFR1 showed its highest occupancy (37.7 %) at day 0. However, from day 7 onward, its occupancy showed a downward trend, decreasing to about 30 %; however, the difference was not significant (*p* > 0.01), and its function was retained until the end of the observation period. VEGFR2 initially showed high occupancy and also showed a downward trend from day 0 to day 28. No significant difference in VEGFR2 occupancy appeared between day 0 (36.1 %) and day 7 (33.5 %), but there was a significant decrease between day 7 and day 14 (24.0 %) (*p* < 0.01); the decrease between day 14 and day 21 (25 %) was not significant (*p* > 0.01), but a significant decrease (*p* < 0.01) was observed between day 21 and day 28 (20.4 %). The occupancy of VEGFR3 increased from day 0 (26.2 %) to day 28, where it represented the majority of VEGFR expression (50.1 %). Its occupancy was lowest at day 0, but it increased significantly from day 0 to day 7 (35.41 %) and to day 14 (47.4 %). No significant difference in VEGFR3 occupancy (*p* > 0.05) appeared from day 14 to day 28, which fluctuated between 45 and 50 %.

The low initial occupancy of VEGFR3 (day 0) suggests that its function at the initial stages of wound healing involves the initial differentiation of the lymphatic vessels along with VEGFR2; however, VEGFR2 occupancy decreased up until day 14. In particular, despite the fact that the occupancy level of VEGFR3 at the end of the observation period (day 28) had increased by about 5 %, there was no significant change in the overall immunoreactivity (pixel number), as the expression of the other VEGFRs decreased. This result suggests that the changes during wound healing are not due to increased VEGFR3 function but rather to a decrease in the functions of the other VEGFRs (particularly VEGFR2, which is in the same signal cascade pathway).

### VEGFR expression during NPWT

**Fig. 4:**
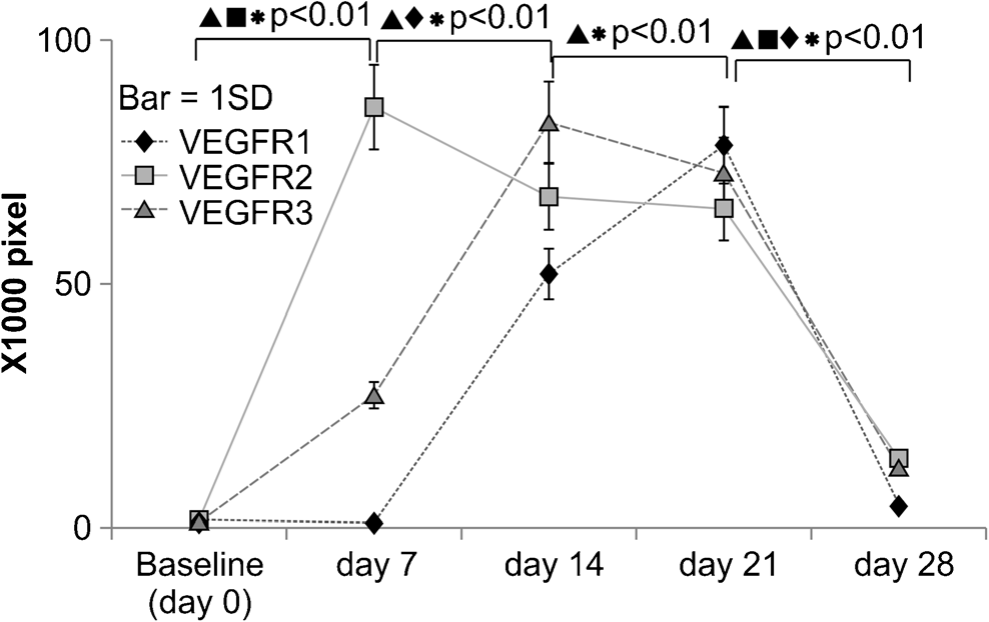
Changes in immunoreactivity in the experimental (NPWT) group from day 0 to day 28. The *x*-axis indicates the day of measurement following wound creation: day 0 (baseline), day 7, day 14, day 21, and day 28. The *y*-axis shows the immunostaining levels of anti-VEGFR1 (*diamonds*), anti-VEGFR2 (*squares*), and anti-VEGFR3 (*triangles*) antibodies, respectively, converted into ×1000 pixels from the captured digital image. The *bars* indicate 1 SD

The absolute values of pixel numbers, indicative of immunoreactivity to an antigen, for all antibodies in the NPWT group are shown in Fig. 4. The mean immunoreactivity of the three VEGFRs at day 0 (baseline level) was 10.2 × 104 pixels and was extremely low on day 28 (0.85 ± 0.12 pixels), representing a decrease of 11.8 % from the mean immunoreactivity on day 21 (7.18 ± 0.85 pixels). This value is comparable to the values observed from day 7 to day 14 in the control group. However, the immunoreactivity to all anti-VEGFR antibodies in the NPWT group was higher than the day-0 values of the control group, although the expression from day 7 until day 21 was reduced considerably. Immunoreactivity to anti-VEGFR2 antibodies showed a peak at day 7 (8.5 ± 0.9 × 104 pixels), representing a significant 5.15-fold increase over the day-0 baseline value (*p* < 0.01) and a significant increase (*p* < 0.01) from the day-14 experimental group (6.7 ± 0.74 × 104 pixels), with subsequent decreases over the course of the experiment. These decreases were only moderately significant (*p* < 0.05), indicating that a certain level of expression was maintained throughout the latter period of wound healing. The immunoreactivity of anti-VEGFR2 antibodies was retained up until day 21. The immunoreactivity of anti-VEGFR3 antibodies also showed a significant 2.2-fold increase (*p* < 0.01) at day 7 (2.5 ± 0.3 × 104 pixels) compared to the day-0 baseline value, although the peak immunoreactivity was observed at day 14 for this VEGFR (8.4 ± 0.94 × 104 pixels), representing a significant 3.36-fold increase from the day-7 value, which subsequently decreased over time. By contrast, the immunoreactivity of the anti-VEGFR1 antibodies decreased significantly (*p* < 0.01) at day 7 (0.26 ± 0.4 × 104 pixels) compared to the day-0 baseline level. There was no significant difference in the immunoreactivity between anti-VEGFR3 and anti-VEGFR2 antibodies on day 14. The immunoreactivity of anti-VEGFR1 antibodies (5.1 ± 0.74 × 104 pixels) showed a significant 20.4-fold increase (*p* < 0.01) at day 7 compared to the baseline, and the rate of increase was largest over day 14.

The staining for all VEGFR antibodies on day 14 was 20.20 ± 3.54 × 104 pixels. No significant difference was observed between the immunoreactivities of the three different kinds of anti-VEGFR antibodies at day 21. The highest immunoreactivity was exhibited by anti-VEGFR1 antibodies (7.8 ± 0.82 × 104 pixels), representing a peak at this point. VEGFR1 was also the only anti-VEGFR antibody to increase significantly (1.47-fold, *p* < 0.01) at day 21 compared to day 14.

All three anti-VEGFR antibodies showed significant drops in immunoreactivity on day 28. The immunostaining of anti-VEGFR1, anti-VEGFR2, and anti-VEGFR3 antibodies decreased by 11.3, 6.92, and 17.24 %, respectively, at the end of the observation period. In particular, the decrease in VEGFR2 and VEGFR1 immunoreactivity suggests that NPWT induces the vascular system. These results also revealed that the expression of VEGFR2 increases during the first stage, and that VEGFR1 and VEGFR3 increased during the second stage of NPWT.

Further, we compared the immunodensity values between the control and experimental groups over the course of the NPWT for all of three VEGFRs (VEGFR1, VEGFR2, and VEGFR3). The immunodensity value in the experimental group was significantly higher than that of the control group at each time point during NPWT (*p* < 0.01). These findings indicate that NPWT has a significant effect on VEGFR expression.

### Changes in the occupancy of each VEGFR during NPWT

**Fig. 5:**
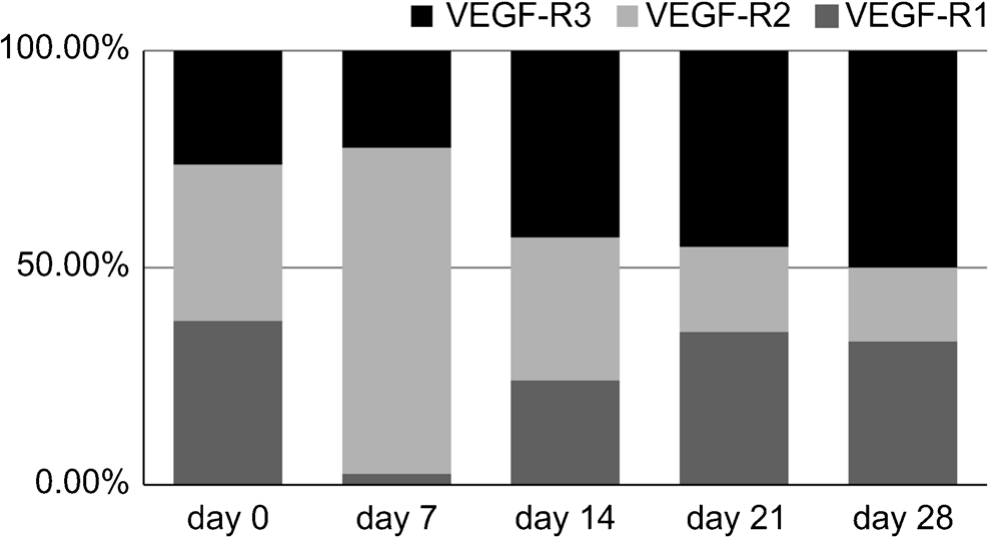
Time course of the occupancy for each VEGFR during negative-pressure wound healing. The *x*-axis indicates the day of measurement after wound creation: day 0 (baseline), day 7, day 14, day 21, and day 28. The *y*-axis shows the relative degree of staining of anti-VEGFR1, anti-VEGFR2, and anti-VEGFR3 antibodies in relation to the total stained area of all VEGFRs to reflect the occupancy (%) of VEGFR1, VEGFR2, and VEGFR3, respectively. These values were converted to ×1000 pixels from the captured digital images

The changes in the occupancy of the three VEGFRs over the course of NPWT are shown in Fig. 5. The VEGFR3 occupancy increased significantly compared to that of day 21, whereas the occupancy of VEGFR2 decreased significantly, and there was no change in the occupancy of VEGFR1 during NPWT.

VEGFR1 showed the lowest occupancy (2.6 %) on day 7 and exhibited an upward trend from day 14 onward, with a 9-fold increase from day 7 to day 14 (24.0 %). However, its highest occupancy value was observed at day 21 (35.2 %), and this state was maintained until day 28 (33.0 %); there was no significant difference (*p* > 0.01) between day 21 and day 28 values. Furthermore, although the occupancy values of VEGFR1 were high at days 21 and 28, they were both lower than the occupancy of VEGFR3 at these time points.

On day 7, the occupancy of VEGFR3 (22.3 %) was lower than that of VEGFR2, but higher than that of VEGFR1. VEGFR3 showed the highest occupancy at days 14, 21, and 28 (43.0, 45.2, and 50.0 %, respectively), with no significant differences between them (*p* > 0.01). This suggests that occupancy itself stabilized in the latter half of NPWT, from day 14 onward, which is also reflected by the fact that the values were higher than those of VEGFR1.

VEGFR2 showed the highest occupancy (75.1 %) of all experimental groups on day 7, which subsequently decreased sharply at day 14 (33.0 %) and day 21 (19.6 %), representing a rate of decrease of 43.9 and 59.4 %, respectively. There was no significant difference in the occupancy between day 21 and day 28 (17.0 %), when VEGFR2 showed the lowest occupancy. These trends confirmed the results of the degree of staining (pixel values) described above.

These results suggest that although the expression of VEGFR3 decreased in terms of its absolute value over the course of NPWT, its occupancy increased. Overall, VEGFR2 and VEGFR3 showed the highest occupancy during the first and second stage of NPWT, respectively.

### Histopathological analysis

**Fig. 6:**
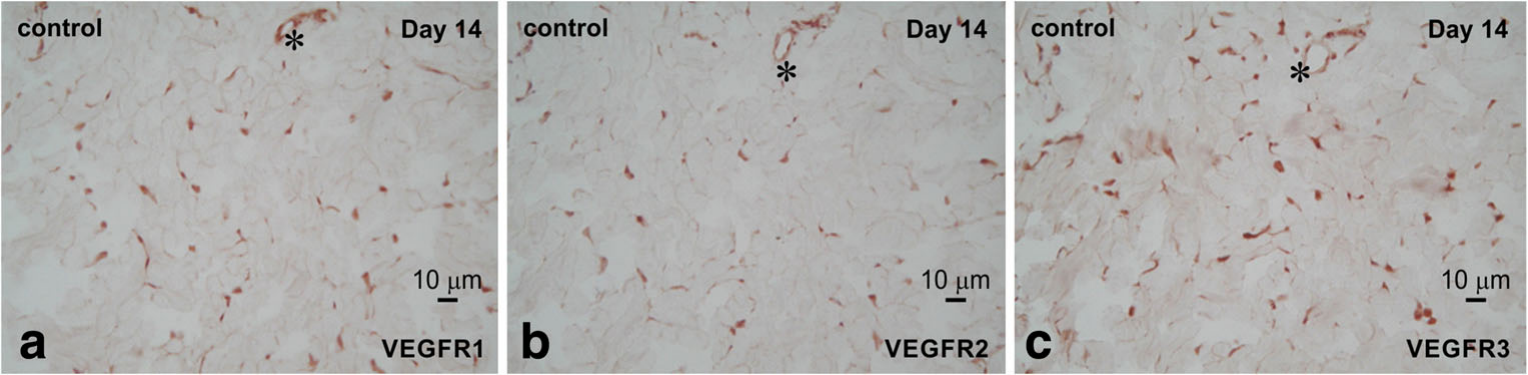
The three adjacent VEGFRs-immunostained sections of the control group at 14 days revealed that the highest and lowest immunodensity/number of cells were observed for VEGFR3 (**c**) and VEGFR2 (**b**), respectively. This immunoreactivity to VEGFRs was weakly and diffusely observed in a portion of the endothelial vessel cells. *, same vessel in (**a)**–**(c)**

**Fig. 7:**
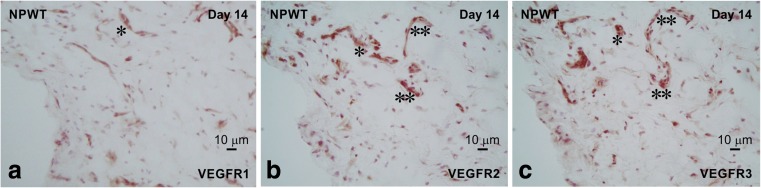
The three adjacent VEGFRs-immunostained sections of the NPWT experimental group at day 14 revealed that the highest and lowest immunodensity/number of cells were observed for VEGFR3 (**c**) and VEGFR1 (**a**), respectively. This immunoreactivity was strongly and widespread completely in a specific vessel-formation area. *, VEGFR1–3 immunoreactive vessels in (**a)**–**(c)**; **, VEGFR2 and VEGFR3 immunoreactive vessels in (**b**) and (**c**), respectively

Figures 6 and 7 show the comparison of immunoreactivity in the localization and formation of VEGFRs between the control groups and NPWT group on day 14.

In the control group, the expression of VEGFR1 and VEGFR3 was strongly detected, but that of VEGFR2 was weak. However, in the NPWT group, the expression of VEGFR2 and VEGFR3 was strongly observed. Thus, the immunoreactivity results corroborated with the quantitative results for VEGFRs expression.

In particular, the localization and formation of VEGFR immunoreactivity in the control group was found to be diffuse and incomplete along the structure of vessels, whereas immunoreactivity in the NPWT group revealed specific localization and a complete structure of vessels. These results suggest that NPWT induced strong immunoreactivity of VEGFRs and indicated that NPWT induced complete vessel formation and construction.

In the NPWT group, the injured wound surfaces (Fig. 1a) were completely covered and vacuumed by the urethane foam included with the VAC systems (Fig. 1b, c). Furthermore, Fig. 1c shows that the surfaces of the wounded holes in the back skin were vacuumed and induced negative pressure by using the NPWT systems. These figures indicate that the surfaces of wounded skin holes disappeared under the negative pressure environment.

## Discussion

### Normal pressure wound healing

VEGFR expression in normal cells was highest for VEGFR1, followed by VEGFR2 and then VEGFR3, indicating that in healthy tissue (day 0). Furthermore, there was very little difference in the expression of the three receptors over time in terms of the overall degree of staining, whereas the occupancy results (proportion of all VEGFRs occupied by a given receptor) showed that the occupancy of VEGFR3 tended to be higher and that of VEGFR1 tended to be lower. From day 7 to day 14, the occupancy value for VEGFR3 increased further, and then the value of VEGFR2 increased thereafter. This result indicates that these upstream cascade reactions undergo changes during this time period. In particular, VEGFR3 is mainly activated by VEGF-C and -D related cascades, whereas VEGFR1 is mainly activated by VEGF-A, as well as VEGF-C and -D; thus, the branching point at days 7 to 14 appears to be represented by decreased activation of VEGF-A. Indeed, VEGF-A is a common activating factor of VEGFR1 and VEGFR2, which could explain the observed shift at day 7 and day 14, with the occupancy of VEGFR1 decreasing significantly on day 7 but that of VEGFR2 dropping to an extreme degree on day 14. In other words, the actions of VEGF-A on VEGFR1 and VEGFR2 predominantly affected the expression of VEGFR2 by day 7, with low occupancy of VEGFR1, but by day 14, the expression of VEGFR1 began to increase sequentially.

The main subsets of VEGF-A are VEGF-A121 and VEGF-A165, and their balance is relevant to VEGFR1 and VEGFR2 activity and expression. Mac Gabhann et al. [24, 25] have stated that the action of VEGFR2 is intensified due to activation of VEGF-A165 in the presence of neuropilin, mainly in the process of muscle differentiation or in the vascular endothelium; in the absence of neuropilin, activation of both VEGF-A165 and VEGF-A125 resulted in more intense activity of VEGFR1 than VEGFR2. These facts suggest that on day 7 in this study, neuropilin was present in abundance in the tissue, and that its levels decreased by day 14. Furthermore, on day 28, VEGFR1 and VEGFR2 expression decreased significantly, but there was no change in the occupancy of VEGFR1 although that of VEGFR2 decreased. This implies that at the period from day 21 to day 28, VEGFR3 had also decreased; therefore, despite an overall change in the activity of the cascade in terms of VEGF-A, -C, and -D as a whole, there may have been a particular change in the balance of VEGFA in which VEGF-A165 might have decreased more than VEGFR-A125 in the absence of neuropilin.

The immunoreactivity of VEGFR3 peaked at day 21 and was significantly higher than the others, with the highest rate of increase. At day 14, there was a significant difference between VEGFR3 and VEGFR2 expression, which is therefore considered the time point representing a change in the VEGF-C and VEGF-D signaling cascades upstream of VEGFR2 and VEGFR3. It is unclear whether this difference is due to the more significant antigenicity of VEGFR3 compared to VEGFR2 or to the actions of VEGF-C and -D. VEGFR3 expression is indicative of differentiation of lymphatic vessel-like vascular epithelial tissues. However, the earlier increase in VEGFR3 on day 14 implies that the antigenicity of VEGFR1 and VEGFR2 was also elevated at this point.

In summary, considering previous findings in light of the present experimental results, we can conclude that under normal circumstances, lymphatic differentiation involves the complete cycle for VEGF-C: VEGFR3 is activated following VEGFR2 activation, and typical lymphatic differentiation takes place. The results of the present study also imply that changes in the antigenicity of VEGFR2 and VEGFR3 might ultimately converge to complete the formation of lymphatic vessels, especially from the aspects of recovery timing in wound healing.

### NPWT

In the experimental group that underwent NPWT, the patterns of the emergence of expression of the VEGFRs were completely different from those in the control group. At day 7, the expression of VEGFR2 already reached its maximum value, followed by high expression of VEGFR3 and low immunoreactivity to anti-VEGFR1. In light of the results of the control, we assumed that activation of VEGF-C and -D had already initiated considerable differentiation of cells, particularly those exhibiting VEGFR2 expression at this time. Moreover, the fact that expression of VEGFR3 was significantly higher than that of VEGFR1 implies that VEGF-A activation had not yet begun. Given that NPWT induces VEGFR and accelerates wound healing, Kieesling et al. [27] suggested that performing NPWT at the time of sternum closure after heart disease surgery would promote wound healing through vascular induction accompanied by cytokine induction. Furthermore, according to Bletsa et al. [28], the binding of VEGF-A to VEGFR2 causes DLL4 induction in vascular tip cells, leading to Notch signaling in vascular endothelial cells to induce VEGFR2 and VEGFR3, thereby accelerating vascular endothelial cell and lymphatic vessel differentiation. Furthermore, VEGFR3, which is a principal receptor of VEGF-C, has been suggested to be more strongly induced by Notch [26].

At days 14–21, all of the VEGFRs were active, and their expression levels were similar on day 21. As indicated above, VEGF-C and VEGF-D are considered common activation factors for all VEGFRs. The stable emergence of VEGFR3 was a particular characteristic feature of this period. Rissanen et al. [29] and Anisimov et al. [30] attempted to use adenovirus as a gene expression vector to induce VEGFR3 in response to external/long-term stress and found that VEGF-D, which acts on CAC base repetitions, likely has the higher contribution to stronger VEGFR3 promotion. In the present study, both VEGFR3 and VEGFR2 maintained high levels of expression continuously throughout the mid- and late stages (days 14–21) of NPWT, suggesting that NPWT induces VEGFR2 expression via a higher ratio of VEGF-C/-D than VEGF-A.

Yamanaka et al. [31] stated that VEGFR2 promotion acts on the blood vessel and lymphatic systems and contributes to wound healing on the wound surface, and Bletsa et al. [28] reported an association of VEGF-D with lymphatic repair ability. The high expression of VEGFR3 observed at the deep wound surface in the present experimental system was observed at days 14–21, with a constant intensity of tissue staining during this period, implying that VEGF-D-derived promotion factors would also be particularly relevant for the deep sites that were highly stainable.

Regarding lymphatic differentiation, our results suggested that differentiation of the blood vessel system was occurring as of day 14, owing to the rapid increase in the immunoreactivity and occupancy of VEGFR1. VEGFR1 expression peaked at day 21 and then declined rapidly on day 28. In contrast to the control group where VEGFR1 maintained its peak position, the peak at 21 days in the NPWT group was transient. Kumar et al. [32] found considerable expression of VEGFR1, VEGFR2, and neuropilin-1 and -2 at the wound surface during postoperative repair from vascular system stress, and only VEGFR1 decreased rapidly after completion. In many aspects, this is consistent with the results of the present study, especially for the results of the control where, in the assumed presence of neuropilins, activation of both VEGF-A165 and VEGF-A125 likely allowed for VEGFR2 to be more readily expressed than VEGFR1, and therefore lowered the antigenicity of VEGFR1.

The fact that all VEGFRs showed abundant expression at day 21 is considered to be induced by the respective VEGF signaling cascades. In particular, the accumulation of VEGF-C exhibits this phenotype from the induction of the vascular system, which is represented by an increase in basic fibroblast growth factor (bFGF). Koolwijk et al. [33] suggested that VEGFR3 is expressed in the lymphatic system, whereas VEGFR1 is expressed in the blood vessel system, and that their abundant and simultaneous expression causes induction of the fibroblast system. In particular, early expression or increased expression levels of VEGFR2 in the NPWT group are believed to help the functioning of fibroblasts that make up the blood vessel system and lymphatic system.

In light of the present experimental results, compared to normal wound healing, the NPWT group showed higher maximum activation values for VEGFR3 and VEGFR2, as well as earlier timing of maximum expression; however, in the case of VEGFR1, the maximum activation value was higher but the timing of maximum expression was the same as the control. Moreover, according to Joukov et al. [34], expression of the Np2 gene and VEGFR3 results in an earlier transition of the timing of the generation of the vascular system in the embryonic stage via tyrosine kinase. In addition, the phenomenon of the earlier expression of VEGFR3 would require participation of a VEGFR3/Np2 activity system, which is a series of reaction systems [35]. Ferrel et al. [36] screened for genes involved in the Np2 activity system in humans and found that the upstream cascade involved in lymphatic regeneration in lymphoma was mainly activated by VEGF-C, which in turn activates VEGFR2 and VEGFR3 that share an Np2 activity system. This same phenomenon could be responsible for the expression patterns observed in the NPWT group, thereby producing an early increase in VEGFR3 based on Np2 as a growth factor of the lymphatic vessels and blood vessels.

In summary, with negative pressure applied to the wound surface, both VEGFR2 and VEGFR3 were expressed abundantly in the early stage in this experimental model, which induced early wound healing.

### Potential mechanism and clinical prospects for NPWT

Erba et al. [3] reported early induction of wound healing due to early high expression of the VEGF-C dimer and a urethane effect. Similarly, in the present experimental system, VEGFR2 levels were abnormally elevated in the NPWT group compared to normal wound healing group on day 7 (the first day of measurement). Incidentally, Erba et al. [3] used a pressure of −125 mmHg, which is the same condition as the VAC treatment system used in our study. Therefore, in a VAC treatment system under similar conditions (−125 mmHg/continuous mode), these results strongly suggest that VEGFR2, VEGFR1, and VEGFR3 appear early and are involved in the differentiation and growth of the vascular system.

Plikaitis et al. [1] found that NPWT was involved in myofibroblast construction after inducing granulation tissue to promote the creation of new blood vessel systems in the wound healing process. In the present experimental system, we immunohistochemically confirmed that expression of VEFGRs was activated on day 21 during normal wound healing, which was completed much earlier with NPWT, supporting the results of previous work.

To investigate the relevance of negative pressure force to wound healing, Zhou et al. [2] created wounds on the backs of Göttingen minipigs and compared various wound-healing markers and the wound-healing area under various levels of negative pressure over time. Bacterial infection significantly decreased under all pressure levels, whereas capillary system regeneration and expression of the capillary proteins and VEGF and bFGF occurred at a relatively earlier stage in the low-pressure groups. The authors concluded that revascularization occurred earlier under NPWT at low pressure (−75 to −150 mmHg) based on the earlier activity of bFGF and VEGF, which promoted the formation and differentiation of granulation tissue.

Considering these previous findings and the results of the present study, the standard clinical use of a negative-pressure level of NPWT of −125 mmHg is regarded as valid. However, further research on different wound-healing states at even earlier stages after surgery (days 1–7) is required, and more detailed experimentation for each day would provide valuable insight into the mechanisms of NPWT, which could help to improve its clinical utility.

Finally, the specific focus of this experiment was to evaluate the reactions of VEGF-A, B, -C, and -D (VEGFR1, R2, and R3) using a rabbit model. However, in the wound-healing experiment, VEGF-F caused epidermal regeneration in NPWT in the same way as observed with VEGF-A to -D. In addition, the use of a porcine model would fundamentally allow for a comparison to human skin, since they have the same structure, and would further enable creation of a larger wound area. Given that one limitation of the study was that the mRNA expression level was not detected, further studies should be carried out using an in situ hybridization method. Thus, future work should focus on understanding the wound-healing mechanism of NPWT using VEGFR and a porcine model, which will likely help in obtaining clearer and more clinically translatable results.
